# Patterns of Disease Progression and Outcomes of Inferior ST-Elevation Myocardial Infarction Complicated by Cardiogenic Shock: The Multicenter INSTINCT Registry

**DOI:** 10.3390/jcm14072231

**Published:** 2025-03-25

**Authors:** Giulia Botti, Marina Pieri, Luigi Cappannoli, Andrea Raffaele Munafò, Mario Gramegna, Marco Gamardella, Rita Camporotondo, Cristina Aurigemma, Marco Ferlini, Stefania Guida, Angelicarosa Cascone, Filippo Russo, Giuseppe Lanzillo, Francesco Burzotta, Matteo Montorfano, Anna Mara Scandroglio, Alaide Chieffo

**Affiliations:** 1 School of Medicine, Vita Salute San Raffaele University, 20132 Milan, Italy; giulia.bottimail@gmail.com (G.B.); gamardella.marco@hsr.it (M.G.); a.cascone2@studenti.unisr.it (A.C.); montorfano.matteo@hsr.it (M.M.); 2 Interventional Cardiology Unit, IRCCS Ospedale San Raffaele, 20132 Milan, Italy; russo.filippo@hsr.it; 3 Department of Cardiothoracic and Vascular Surgery, IRCCS San Raffaele Scientific Institute, 20132 Milan, Italy; pieri.marina@hsr.it (M.P.); gramegna.mario@hsr.it (M.G.); scandroglio.mara@hsr.it (A.M.S.); 4 Department of Cardiovascular Sciences, Fondazione Policlinico Universitario A. Gemelli IRCCS, 00136 Rome, Italy; luigi.cappannoli@gmail.com (L.C.); aurigemma.cristina@gmail.com (C.A.); francescoburzotta@gmail.com (F.B.); 5 SC Cardiologia1, Fondazione IRCCS Policlinico San Matteo, 27100 Pavia, Italy; andrearaffaele.munafo01@universitadipavia.it (A.R.M.); r.camporotondo@smatteo.pv.it (R.C.); m.ferlini@smatteo.pv.it (M.F.); s.guida@smatteo.pv.it (S.G.); g.lanzillo@smatteo.pv.it (G.L.); 6 De Gasperis Cardio Center, Interventional Cardiology Unit, Niguarda Hospital, 20162 Milan, Italy

**Keywords:** cardiogenic shock, inferior STEMI, prognosis, in-hospital outcomes

## Abstract

**Background**: Cardiogenic shock (CS) is a frequent presentation of anterior ST-elevation myocardial infarction (STEMI); however, data regarding disease progression and outcomes in inferior STEMI complicated by CS are scarce. The present study aims to analyze the prevalence, patterns of disease progression, and outcomes of inferior STEMI-CS. **Methods**: The INSTINCT (Inferior ST-elevation myocardial Infarction complicated by Cardiogenic shock) Registry retrospectively included consecutive patients who developed CS following inferior STEMI treated at three centers in Italy from 2015 to 2023. Data regarding CS stage according to the Society of Cardiovascular Angiography and Interventions (SCAI) upon diagnosis of shock and during disease progression and in-hospital outcomes were collected. Patients were defined “worsening” (WPs) if the SCAI stage increased. **Results**: A total of 130 patients developed CS after inferior STEMI and were included in the analysis, the mean age was 69.8 ± 12.4 years, and 31.5% were female. The rate of in-hospital mortality was 22.3%; predictors of in-hospital mortality were cardiopulmonary resuscitation (CPR) > 20 min or refractory cardiac arrest (CA) (OR [CI]: 9.67 [3.17–29.51]), persistently low systolic blood pressure (SBP) (OR [CI]: 12.91 [2.47–68.82]), and increase in lactates (OR [CI]: 3.53 [1.42–7.87]) during medical management. Twenty (15.4%) patients experienced worsening CS; WPs had a significantly higher rate of in-hospital mortality (13 [65%] vs. 15 [13.6%], *p* < 0.001), major bleeding (4 [20%] vs. 7 [6.4%], *p* = 0.044), and mechanical circulatory support weaning failure (7 [35%] vs. 3 [2.7%], *p* = 0.032). **Conclusions**: The in-hospital mortality rate of inferior STEMI complicated by CS was 22.3%. Predictors of in-hospital mortality included prolonged CPR, persistently low SBP, and elevated lactates. Progression through SCAI stages was rare but associated with significantly higher mortality and complication rates.

## 1. Introduction

Despite continuous improvements in the prompt management of ST-elevation myocardial infarction (STEMI), cardiogenic shock (CS) remains a frequent clinical presentation of anterior STEMI [1]. The incidence of acute coronary syndromes (ACS) complicated by CS is reported to be between 4% and 12% [1]. Notably, CS is the condition at presentation in 30–40% of cases, whereas up to 70% of patients develop CS during hospitalization [2]; moreover, up to 90% of patients who present at the initial stages of CS experience in-hospital worsening of the clinical picture [3]. Most importantly, although a decrease in the incidence of CS complicating ACS has been reported in recent decades, the mortality rate remains high, unchanged (40–50%), and more prevalent among patients who develop CS during their hospital stay [4]. To date, CS remains the main cause of in-hospital mortality associated to myocardial infarction [5].

Patients who suffer inferior STEMI are considered to have a better prognosis compared to anterior STEMI, even when the clinical picture is complicated by cardiogenic shock (CS) [6]. However, right ventricular (RV) failure cannot be defined as a benign condition, as it is the leading cause of morbidity and mortality in patients with advanced heart failure (HF), pulmonary hypertension, and acute myocardial infarction following cardiac surgery [7]. Overall, data focusing specifically on inferior STEMI with RV involvement and RV-predominant CS are scarce, since most studies focus on left ventricular (LV)-predominant CS, which represents the majority of STEMIs complicated by CS [3,8].

Therefore, the present study aims to explore the prevalence of inferior STEMI complicated by CS, analyze the patterns of disease progression during patient management in intensive care settings, and investigate in-hospital outcomes.

## 2. Materials and Methods

### 2.1. Study Population

**Figure 1:**
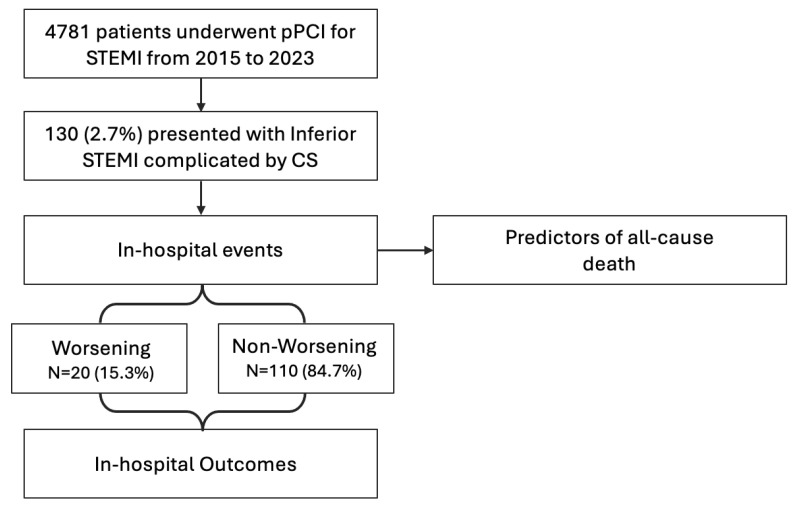
Flowchart of the study. CS: cardiogenic shock; pPCI: primary percutaneous coronary intervention; RV: right ventricle; and STEMI: ST-elevation myocardial infarction.

The INSTINCT (Inferior ST-elevation myocardial Infarction complicated by Cardiogenic shock) Registry is a retrospective multicenter observational national registry that includes all consecutive patients who developed CS following inferior STEMI treated at three high-volume tertiary-level centers in Italy (IRCCS Ospedale San Raffaele, Milan; IRCCS Policlinico Gemelli, Rome; IRCCS Policlinico San Matteo, Pavia, Italy) from 2015 to 2023. The study flowchart is displayed in Figure 1.

The total number of patients with STEMI undergoing primary PCI was obtained from the annual reports of the Italian Society of Interventional Cardiology (GISE) to provide an epidemiological perspective, while the study population was identified from individual hospital records. Data on medical history, clinical presentation, procedural characteristics, in-hospital management, and in-hospital outcomes were collected in a prespecified dataset prior to adequate anonymization; therefore, the final analyses were performed on an anonymized set of aggregate data.

### 2.2. Definitions and Outcomes

Patients with acute thrombotic occlusion of the proximal or mid segment (syntax segments 1 and 2 [9]) of a dominant right coronary artery and CS were included in the analysis. CS was defined according to the Cardiogenic Shock Working Group-defined Society of Cardiovascular Angiography and Interventions (CSWG-SCAI) staging [3,10] and patients with SCAI stage B to E were included. More specifically, Table S1 describes the specific definitions used for each SCAI stage.

Since cardiac arrest has been identified as a disease modifier [3], patients presenting with cardiogenic shock requiring CPR for more than five minutes or developing refractory cardiac arrest were attributed a SCAI stage E, irrespective of other criteria. Conversely, patients who experienced return of spontaneous circulation (ROSC) after <5 min of CPR and those who suffered a periprocedural reperfusion arrythmia that was successfully reversed were stratified according to the described criteria. Patients were defined as “worsening” if the CSWG-SCAI stage increased during in-hospital management.

Due to the retrospective nature of this study, a series of adaptations were necessary to collect the data in a reproducible fashion across all involved centers. To describe patient characteristics at diagnosis of shock, the first available parameters or markers were collected, whereas data in cases of clinical improvement or deterioration were reported to describe disease progression. Depending on the availability of data, both parameters at discharge from the ICU or hospital were considered acceptable to investigate in-hospital outcomes. Regarding in-hospital events, major bleeding was defined as any bleeding ≥Type 2 according to the bleeding academic research consortium (BARC) [11], while bleeding or ischemic complications derived from the positioning of a mechanical circulatory support device were considered device access site complications. Right ventricular dysfunction was defined as TAPSE < 17 mm and/or S’TDI < 9 cm/s and biventricular dysfunction was reported in case of concomitant right ventricular dysfunction and LVEF < 50%. PCI failure was defined as a thrombolysis in myocardial infarction (TIMI) flow grade ≤ 2. In accordance with the SCAI classification framework, particularly as described by the Cardiogenic Shock Working Group, SBP < 90 mmHg and <60 mmHg and lactate > 5 mmol/L and >10 mmol/L were selected as cutoff points to categorize hemodynamic impairment and metabolic dysfunction [3,10].

The present study aimed to analyze the patterns of disease progression (i.e., deterioration of SCAI stage) in inferior STEMI complicated by CS and evaluate in-hospital outcomes, including all-cause death, major bleeding, sepsis, and failure to wean from mechanical circulatory support (MCS).

### 2.3. Statistical Analysis

Continuous variables were reported as the mean ± standard deviation or median (interquartile range) and were compared using Student’s *t*-test or the Mann–Whitney U or Wilcoxon test, as appropriate. Categorical variables were reported as a number (percentage) and were compared using the chi-square test or the Fisher exact test, as appropriate. ANOVA for repeated measures and Freidman test were used to compare characteristics at baseline and disease progression. In-hospital outcomes were analyzed using logistical regression analysis, a 95% confidence interval was considered, and a two-sided *p*-value < 0.05 defined statistical significance. Predictors of in-hospital death were estimated with multivariate logistic regression models, including the most relevant variables from a clinical and pathophysiological point of view, with a *p*-value < 0.10 at univariate analysis and using a rule of 1:10 covariates per number of events to avoid overfitting. The analysis was conducted using SPSS version 29.0 (IBM, Armonk, NY, USA).

## 3. Results

### 3.1. Study Population and Clinical Presentation at Diagnosis of Shock

Out of 4871 patients who underwent primary PCI for STEMI at the three centers from 2015 to 2023, 130 met the criteria to be included in the analysis. The mean age was 69.8 ± 12.4 years, and 41 (31.5%) were female. Baseline characteristics and clinical presentation are summarized in Table 1. A total of 68 (52.3%) patients were smokers, 85 (65.4%) suffered from hypertension, 39 (30.0%) from diabetes, and 38 (29.2%) from chronic kidney disease (CKD). A total of 42 (32.3%) patients presented with out-of-hospital cardiac arrest (OHCA), 25 (19.2%) required <20 min of cardiopulmonary resuscitation (CPR) maneuvers, 9 (6.9%) required prolonged (>20 min) CPR, whereas 8 (6.2%) experienced refractory cardiac arrest.

Table 2 describes laboratory and echocardiographic workup upon diagnosis of cardiogenic shock. Patients presented with increased mean lactates (3.65 [2.6–5.8] mmol/L) and glucose (220.15 ± 106.52 mg/dL), whereas mean creatinine (1.08 [0.9–1.5] mg/dL) and blood gas parameters (SpO2: 98.75 ± 40.71%, PCO2: 39.44 ± 13.79 mmHg, HCO3^−^: 20.29 ± 5.11 mEq/L, pH: 7.32 ± 0.15) were not relevantly altered. At echocardiography upon diagnosis of shock, patients had a mildly reduced mean left ventricular ejection fraction (LVEF 43.17 ± 11.1%), right ventricle dysfunction (RVD) (TAPSE 14.03 ± 4.68 mm, S’TDI 8.26 ± 3.61 cm/s), an increased systolic pulmonary artery pressure (PAPs 34.69 ± 9.06 mmHg), and a dilated inferior vena cava (IVC 20.14 ± 4.40 mm). Biventricular dysfunction was detected in 44 (33.8%) patients.

Details regarding in-hospital management are reported in Table 3. Primary percutaneous coronary intervention (pPCI) was performed in all patients; 83 (63.8%) were affected by multivessel coronary artery disease (CAD) and 12 (9.2%) by left main CAD. Concomitant multivessel PCI was performed in 19 (14.6%) cases, and all patients who displayed severe LM disease were treated, except for 2 patients for whom pPCI failed. Prompt support with inotropic medication was required in 99 (76.2%) patients, 40 (30.8%) received mechanical ventilation, and 49 (37.8%) received MCS. More specifically, venoarterial membrane oxygenation (VA-ECMO) was used in 9 (6.9%) patients, intra-aortic balloon pump (IABP) in 41 (31.5%), Impella for the left ventricle (CP or 2.5) in 9 (6.9%), and Impella RP in 9 (6.9%). Furthermore, 19 (14.6%) patients received renal replacement therapy, and 30 (23.1%) underwent blood transfusion. Successful weaning from MCS was possible in 39 out of the 49 patients who received MCS (79.5% of patients who received MCS).

Table 4A,B describes the progression of laboratory and hemodynamic parameters from diagnosis of shock throughout the progression of the disease. Peak laboratory values were significantly higher than those at diagnosis of shock, whereas the hemodynamic parameters improved steadily upon the initiation of treatment for CS.

### 3.2. In-Hospital Outcomes and Predictors of All-Cause Mortality

In-hospital outcomes are displayed in Table 5. The in-hospital mortality rate was 22.3% (29 patients), 11 (8.5%) patients suffered major bleeding events, device access site complications and hemolysis were reported in 6 (4.6%) and 8 (6.2%) patients, respectively, and 21 (16.2%) patients developed sepsis. Baseline characteristics comparing survivors and non-survivors are displayed in Table S2.

The median duration of both inotropic and mechanical circulatory support was 3.0 [2.0–5.0] days; the median duration of ICU and hospital stay was 4.5 [3.0–8.0] and 10.0 [7.0–15.3] days, respectively. Regarding echocardiographic parameters at discharge, LVEF improved to 48.2 ± 10.6%, TAPSE recovered to 17.9 ± 4.2 mm, and the right ventricle diameter 1 (RVD1) decreased to 33.3 ± 8.9 mm. Residual at least moderate tricuspid regurgitation was present in 11 (8.5%) patients, and 26 (20.0%) patients were discharged with a residual RV dysfunction.

Overall, cardiac arrest at presentation was not associated with an increased risk of all-cause death (survivors: 30 [29.7%] vs. non-survivors: 12 [41.4%], OR [CI]: 1.69 [0.72–3.98]); however, refractory cardiac arrest and cardiac arrest requiring CPR for over 20 min significantly increased the odds ratio for in-hospital mortality (survivors: 11 [10.9] vs. non-survivors: 6 [20.7], OR [CI]: 9.67 [3.17–29.51]). Non-survivors also received more frequent mechanical ventilation (14 [48.3%] vs. 26 [25.7%], OR [CI]: 3.28 [1.34–8.0]). Worsening laboratory values and hemodynamic characteristics, rather than more deteriorated clinical pictures at diagnosis of shock, increased the odds ratio for mortality (SBP < 90 mmHg at progression: 2 [2.0] vs. 6 [20.7], OR [CI]: 12.91 [2.47–68.82]; increasing lactates at progression: 21 [20.5] vs. 7 [24.1], OR [CI]: 3.53 [1.42–7.87]). Finally, patients who did not survive had a lower mean TAPSE at diagnosis of shock. The results are reported in Table 6.

**Figure 2:**
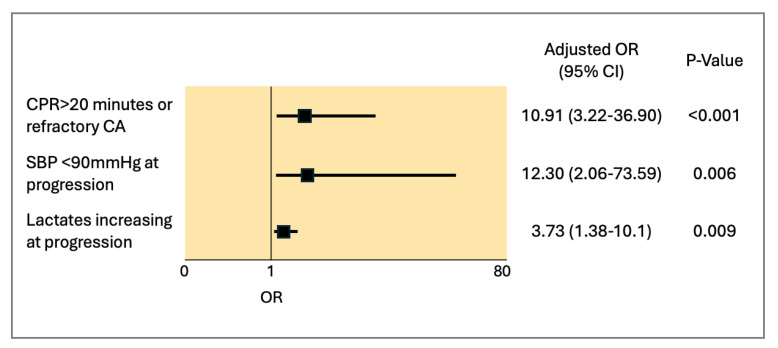
Multivariate analysis for in-hospital predictors of all-cause death. CPR: cardiopulmonary resuscitation; CA: cardiac arrest; and SBP: systolic blood pressure.

In the multivariate analysis, which was performed including CPR > 20 min or refractory CA and SBP < 90 mmHg, despite medical management and increasing lactates, all variables remained independent predictors of in-hospital mortality. These results are shown in Figure 2.

### 3.3. Patterns of Progression of Cardiogenic Shock

**Figure 3:**
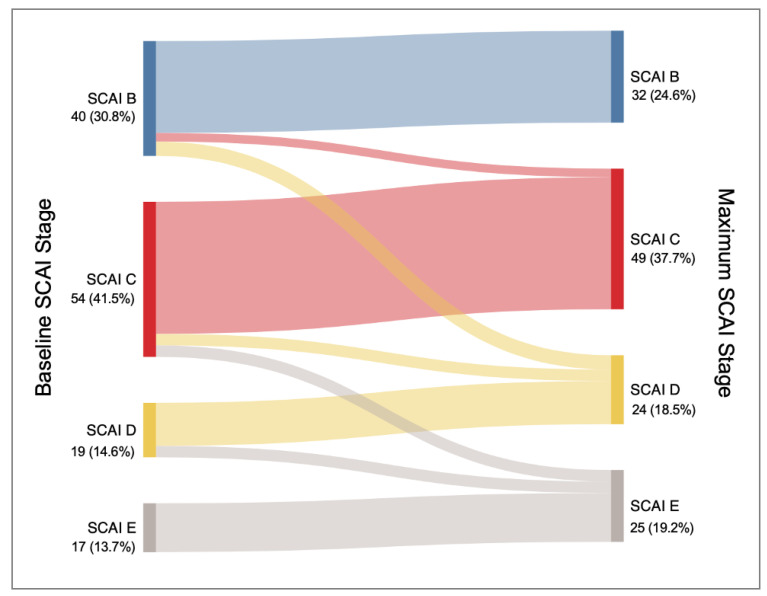
SCAI stages at baseline and progression patterns towards the maximum reported SCAI stage. SCAI: Society for Cardiovascular Angiography and Interventions.

Upon admission, 40 (30.8%) patients were attributed SCAI B stage, 54 (41.5%) SCAI C, 19 (14.6%) SCAI D, and 17 (13.7%) SCAI E. Figure 3 shows that most patients did not experience a worsening of the SCAI stage, which was limited to 20 (15.3%) patients. However, 85% (17/20) of patients who showed in-hospital worsening progressed towards severe stages of CS (SCAI D or E). The two groups were comparable regarding baseline characteristics, as displayed in Table S3.

Worsening of the SCAI stage was associated with PCI failure (10 [50%] vs. 17 (15.4%), OR [CI]: 5.41 [1.96–14.97]) and worsening of metabolic markers such as lactates, creatinine, AST, and ALT, as described in Table 7. The use of VA-ECMO and Impella for the left ventricle was also associated with worsening patterns, whereas the use of Impella RP was not.

**Figure 4:**
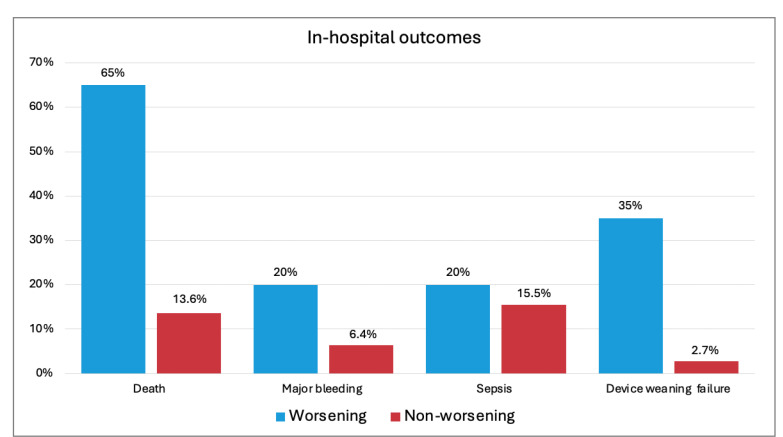
In-hospital outcomes for patients experiencing worsening CS and for those who did not change SCAI stage.

Overall, patients who experienced a worsening of SCAI stage had a significantly higher risk of in-hospital death (13 [65%] vs. 15 [13.6%], OR [CI]: 11.63 [4.0–33.87]), major bleeding (4 [20.0%] vs. 7 [6.4%], OR [CI]: 3.70 [2.2–14.0]), and failure to achieve weaning from MCS. The results are available in Figure 4.

## 4. Discussion

The main findings of this study are the following:

1. The prevalence of inferior STEMI complicated by cardiogenic shock among patients undergoing pPCI was low (2.7%). In-hospital mortality reached 22.3%, and complication rates of bleeding, device access site issues, and sepsis were not negligible, being, respectively, 8.5%, 4.6%, and 16.2%.
2. One-third of patients presented with cardiac arrest (CA), and, although CA alone was not a predictor of mortality, prolonged CPR (>20 min) or refractory CA significantly increased the odds. Other predictors included persistently low systolic blood pressure during management and elevated lactate levels.
3. Deterioration in CSWG-SCAI stage, although rare, was associated with higher risks of mortality and complications.

To the best of our knowledge, our study is the first to investigate the in-hospital outcomes and deterioration patterns of inferior STEMI complicated by CS according to the CSWG-SCAI staging system.

The prevalence of inferior STEMI complicated by CS among patients undergoing primary percutaneous coronary intervention (pPCI) is relatively low, at 2.7%; this epidemiological consideration highlights the rarity of the condition and emphasizes the need for collaborative research on this scarcely represented population. Nevertheless, the clinical impact is significant, with an in-hospital mortality rate of 22.3%, which is in the lower limit of mortality rates described in the literature for CS [12,13]. It might be argued that inferior STEMI generally has a better prognosis than anterior STEMI; however, the patients in the present study were included according to the CSWG-SCAI criteria for CS, which are based on the symptomatic and hemodynamic effects and do not account for the underlying etiology. When comparing these results to those from the most recent RCTs on AMICS, it must be noted that the latter often do not include SCAI B patients; still, the mortality rate in our study was lower than that in the CSWG-SCAI validation document by Kapur et al. [3] (22% vs. 35%). These findings suggest that, despite comparable clinical presentations (i.e., same SCAI stages), the mortality of a population with inferior STEMI who develops CS may be lower than that of a population with predominantly anterior STEMI; nevertheless, further studies are needed to confirm this observation. Although this information is poorly investigated, some concordant evidence is also available in literature [6]. The development of CS in patients with inferior STEMI is largely due to right ventricular involvement and congestion leading to hypoperfusion, rather than mechanical complications or severe left ventricular disfunction. Inferior STEMI carries a lower risk of progressing to cardiogenic shock, and, when it does, it often has better outcomes compared to anterior STEMI. Further supporting the aforementioned considerations, in our study population, patients who died in hospital also had a lower TAPSE; however, this information should be interpreted cautiously, since both groups presented a TAPSE below the cutoff of normality (14.5 ± 4.6 vs. 11.6 ± 4.2), and the small sample size did not allow for further analyses in this subgroup. TAPSE < 14 mm has been associated with poor outcomes in patients with chronic heart failure [14], whereas correlations with outcomes in acute settings are less investigated. This finding, however, might suggest that both the involvement and the degree of involvement of the right ventricle (RV) are crucial in determining the outcome of these patients.

Approximately one-third of the patients presented with cardiac arrest; this rate is discordant throughout available studies on CS [12,13]. Moreover, cardiac arrest alone did not necessarily lead to a higher mortality in this population. An increased risk of mortality was observed only when CPR lasted more than 20 min or in case of refractory cardiac arrest. These events in the study population were quite rare (13.7%) and occurred less frequently than reported in the literature for similar events following anterior STEMI complicated by cardiogenic shock [15]. This discrepancy may have contributed positively to outcomes and could explain the observed lower mortality rate. Other predictors of in-hospital mortality were persistently low SBP and increasing lactates despite medical management, whereas severely depressed SBP at presentation (<60 mmHg) and lactates >5 mmol/L were not. This finding might suggest that the crucial aspect in determining the prognosis of this category of patients is in-hospital deterioration rather than pre-existing comorbidities or severe clinical pictures at presentation. Another notable aspect in our population is the relationship between hemodynamic parameters and markers of organ damage. Upon diagnosis of shock, the mean SBP was 87.7 ± 20.4 mmHg, 76.2% received vasoactive therapy, and 37.8% were supported with MCS. The hemodynamic parameters improved steadily during patient management, suggesting a positive response to the inotropic–vasoactive and mechanical support, whereas most cardiometabolic indexes reached a significantly higher peak compared to the measurements at diagnosis of shock.

This finding raises the hypothesis that, while available management options appear effective in stabilizing hemodynamics, end-organ congestion and damage may play a key role in determining outcomes, highlighting potential areas for future therapeutic strategies [16]. In this perspective, efforts are being made to investigate alternative markers for RV failure via targeted multimodal analyses including cardiac magnetic resonance [17], as well as to understand underlying cellular and molecular mechanisms [18] and select the best combination of treatment options [19,20]. Notably, although most patients received inotropic support, the mean vasoactive–inotropic score, which allows one to evaluate the actual load of inotropes the patient is receiving rather than solely the number of medications [21], remained low (5.0 [3.0–10.0] at the beginning of treatment), which underscores the limited medication load which was used in most of the patients. Managing vasoactive therapy in this scenario represents a challenge. Firstly, the choice of the most suitable medication or combination of drugs might be controversial, especially in the setting of biventricular shock, in which some treatments of choice for LV failure might be detrimental on RV function [22]; moreover, almost 40% of patients received mechanical circulatory support, the use of which encourages de-escalation of inotropes [7]. The evidence on the use of MCS in the setting of AMICS has proven its positive impact on reduction in mortality [13]; still, once again, the patients enrolled in these studies are predominantly affected by anterior STEMI complicated by CS. Surely, patients who suffer inferior STEMI with RV involvement should be considered for MCS, accounting for the different pathophysiology of hemodynamic deterioration in order to provide the most targeted and effective approach [7].

It is acknowledged that deteriorating patterns of CS are associated with worse outcomes [23,24]. Moreover, it has been shown that up to 90% of patients who present with apparently benign stages of CS (i.e., SCAI stages B and C) evolve to more severe stages and have a worse prognosis [3]. In our study population, the rate of progression through CSWG-SCAI stages was lower than that reported in the literature. Only 15% of patients progressed to a worse CSWG-SCAI stage during hospitalization. This finding might suggest that patients who develop CS after inferior STEMI tend to achieve rapid stabilization and are not prone to deterioration, if adequately managed. However, patients who experienced deterioration of CS escalated towards advanced and high-risk SCAI stages (85% to SCAI D or E) and had a very poor prognosis. Although these outcomes are merely exploratory and descriptive due to the small number of events, which limited analyses to univariate testing, deteriorating patients developed a severe cardiometabolic shock and required more frequent and aggressive use of MCS. Nevertheless, they experienced more frequent in-hospital complications and mortality. Interestingly, out of the nine patients who received Impella RP devices, eight did not escalate and none died. Although the small number of patients precludes definitive conclusions, this observation highlights the need for further exploration of tailored therapeutic approaches to optimize outcomes while mitigating the risks associated with invasive support devices [25]. Overall, the low progression rate and the poor outcomes of deteriorating clinical pictures highlight the importance not only of the early recognition of CS but also of adequate phenotypization via meticulous monitoring, especially of early-stage and apparently benign presentations. This multifaceted and dynamic approach to CS management might contribute to further lowering the mortality rate in inferior STEMI complicated by CS.

### Study Limitations

This study had several limitations. The retrospective design limited causal conclusions, suggesting that these findings should be regarded as hypothesis-generating. Our study included only patients with inferior STEMI due to right coronary artery occlusion. This methodological choice ensured a more homogeneous population, particularly given our focus on cardiometabolic outcomes, which are more relevant in the setting of right ventricular involvement. Incomplete data on echocardiography and invasive monitoring restricted certain analyses. The use of MCS in some patients might have introduced confounding device-related outcomes such as increased bleeding risks, which are known to worsen the prognosis of these patients [26]; however, the limited number of patients precluded the possibility to perform adjustments. Finally, the small sample size reduced the strength of the outcomes. Given the rarity of this clinical scenario, larger multicenter studies are needed to confirm these results.

## 5. Conclusions

This study confirmed that the prevalence of inferior STEMI complicated by cardiogenic shock is low, with an in-hospital mortality rate of 22.3%. In our study, predictors of in-hospital mortality included prolonged CPR, persistent low systolic blood pressure, and elevated lactate levels. Although progression through CSWG-SCAI stages was rare amongst our patients, such worsening patterns were associated with higher mortality and complication rates.

## Figures and Tables

**Table 1:** Baseline characteristics and clinical presentation.

	*n* = 130
Baseline Characteristics	
Female gender	41 (31.5)
Age	69.8 ± 12.4
BMI	25.5 ± 3.8
Smoker	68 (52.3)
Diabetes	39 (30.0)
Hypertension	85 (65.4)
CAD	20 (15.4)
Heart failure	4 (3.1)
Previous AMI	13 (10.0)
Previous CABG	7 (5.4)
Previous PCI	16 (12.3)
Atrial fibrillation	11 (8.5)
COPD	17 (13.1)
CVA	11 (8.5)
CKD	38 (29.2)
Dialysis	4 (3.1)
Clinical presentation	
Cardiac arrest	42 (32.3)
CPR < 20 min	25 (19.2)
CPR < 20 min	9 (6.9)
Refractory CA	8 (6.2)
SBP (mmHg)	87.7 ± 20.4
DBP (mmHg)	53.4 ± 13.7

BMI: body mass index; CAD: coronary artery disease; AMI: acute myocardial infarction; CABG: coronary artery bypass graft; PCI: percutaneous coronary intervention; COPD: chronic obstructive pulmonary disease; CVA: cerebrovascular accident; CKD: chronic kidney disease; CPR: cardiopulmonary resuscitation; SBP: systolic blood pressure; and DBP: diastolic blood pressure.

**Table 2:** Laboratory workup, blood gas, and echocardiography at diagnosis of shock.

	*n* = 130
Laboratory workup	
Creatinine (mg/dL)	1.08 [0.9–1.5]
BUN (mg/dL)	40 [28–57]
Leucocytes (cells/mm^3^)	14,950 [11,155–18,660]
Platelets (cells/mm^3^)	249,417 ± 95,258
Hemoglobin (g/dL)	13.30 ± 2.30
AST (mU/mL)	106 [40–216]
ALT (mU/mL)	42 [22–83]
Bilirubin (mg/dL)	0.57 [0.35–0.90]
CRP (mg/dL)	4.4 [1.3–40.7]
Blood gas	
SpO_2_ (%)	98.75 ± 40.71
PCO_2_ (mmHg)	39.44 ± 13.79
HCO_3_^−^ (mEq/L)	20.29 ± 5.11
pH	7.32 ± 0.15
Lactates (mmol/L)	3.65 [2.6–5.8]
Glucose (mg/dL)	220.15 ± 106.52
Echocardiography	
LVEF (%)	43.17 ± 11.10
RVD1 (mm)	37.5 ± 6.96
TAPSE (mm)	14.03 ± 4.68
S’ TDI (cm/s)	8.26 ± 3.61
PAPs (mmHg)	34.69 ± 9.06
IVC (mm)	20.14 ± 4.40
Biventricular Dysfunction	44 (33.8)

BUN: blood urea nitrogen; AST: aspartate aminotransferase; ALT: alanine transaminase; CRP: c-reactive protein; LVEF: left ventricular ejection fraction; RVD1: right ventricular diameter 1 (basal); TAPSE: tricuspid annular plane systolic excursion; S’TDI: S’ tissue Doppler imaging; PAPs: systolic pulmonary artery pressure; and IVC: inferior vena cava.

**Table 3:** In-hospital management.

	*n* = 130
PCI	130 (100)
Multivessel disease	83 (63.8)
Left main	12 (9.2)
Left anterior descending	75 (57.7)
Circumflex	50 (38.5)
Concomitant multivessel PCI	19 (14.6)
Inotropes	99 (76.2)
Mechanical ventilation	40 (30.8)
Mechanical circulatory support	49 (37.8)
VA-ECMO	9 (6.9)
IABP	41 (31.5)
Impella LV	9 (6.9)
Impella RP	9 (6.9)
CVVH/Dialysis	19 (14.6)
Cytosorb	7 (5.4)
Blood transfusion	30 (23.1)
Successful device weaning/explantation	39 (30.0)

PCI: percutaneous coronary intervention; VA-ECMO: venoarterial extracorporeal membrane oxygenation; IABP: intra-aortic balloon pump; and CVVH: continuous venovenous hemofiltration.

**Table 4:** Progression of laboratory (A) and hemodynamic (B) parameters.

A	At Diagnosis of Shock		Peak	*p*-Value	
Lactates (mmol/L)	3.65 [2.6–5.8]		4.1 [2.6–7.3]	<0.001	
Creatinine (mg/dL)	1.08 [0.9–1.5]		1.24 [0.9–2.3]	<0.001	
AST (mU/mL)	106 [40–216]		238 [165–401]	0.007	
ALT (mU/mL)	42 [22–83]		70 [41–131]	<0.001	
Bilirubin (mg/dL)	0.57 [0.35–0.90]		0.9 [0.5–1.3]	0.027	
** B **	**At Diagnosis of Shock**	**Start Treatment**	**Progression**	**Recovery/last Available**	***p*** **-Value**
SBP (mmHg)	87.7 ± 20.4	111.23 ± 21.1	116.0 ± 10.1	116.2 ± 20.5	<0.001
DBP (mmHg)	53.4 ± 13.7	59.7 ± 12.0	62.0 ± 11.9	64.2 ± 13.2	<0.001
Inotropic score	-	5.0 [3.0–10.0]	3.0 [.0–6.9]	0	<0.001
Lactates (mmol/L)	3.65 [2.6–5.8]	3.2 [1.9–5.7]	1.94 [1.1–3.2]	1 [0.8–1.7]	<0.001
CVP (mmHg)	-	11.6 ± 5.6	10.4 ± 4.3	8.8 ± 5.3	<0.001

AST: aspartate aminotransferase; ALT: alanine transaminase; SBP: systolic blood pressure; DBP: diastolic blood pressure; and CVP: central venous pressure.

**Table 5:** In-hospital outcomes.

	*n* = 130
Death	29 (22.3)
Major bleeding	11 (8.5)
Device access site complication	6 (4.6)
Hemolysis	8 (6.2)
Stroke	3 (2.3)
Sepsis	21 (16.2)
Duration of inotropic support (days)	3.0 [2.0–5.0]
Duration of MCS (days)	3.0 [2.0–5.0]
ICU stay (days)	4.5 [3.0–8.0]
Hospital stay (days)	10.0 [7.0–15.3]
Echocardiography at recovery/discharge	
LVEF (%)	48.2 ± 10.6
RVD1 (mm)	33.3 ± 8.9
TAPSE (mm)	17.9 ± 4.2
S’ TDI (cm/s)	10.8 ± 2.6
Tricuspid regurgitation (≥2/4)	11 (8.5)
PAPs (mmHg)	30.1 ± 8.7
IVC (mm)	15.1 ± 5.5
Residual RV dysfunction	26 (20.0)

MCS: mechanical circulatory support; ICU: intensive care unit LVEF: left ventricular ejection fraction; RVD1: right ventricular diameter 1 (basal); TAPSE: tricuspid annular plane systolic excursion; S’TDI: S’ tissue Doppler imaging; PAPs: systolic pulmonary artery pressure; IVC: inferior vena cava; and RV: right ventricle.

**Table 6:** Predictors of in-hospital all-cause death.

	Survivors *n* = 101	Non-Survivors *n* = 29	OR (CI)	*p*-Value
Cardiac arrest	30 (29.7)	12 (41.4)	1.69 (0.72–3.98)	0.226
CPR < 20 min or Refractory CA	11 (10.9)	6 (20.7)	9.67 (3.17–29.51)	**<0.001**
Mechanical ventilation	26 (25.7)	14 (48.3)	3.28 (1.34–8.0)	**0.009**
PCI failure	14 (13.9)	13 (44.8)		
Multivessel disease	64 (63.3)	18 (62.1)	0.93 (0.39–2.18)	0.870
SBP				
<90 (mmHg) baseline	56 (55.4)	15 (51.7)	0.84 (0.37–1.93)	0.691
<60 (mmHg) baseline	9 (8.9)	5 (17.2)	2.15 (0.66–7.02)	0.203
<90 (mmHg) at progression	2 (2.0)	6 (20.7)	12.91 (2.47–68.82)	**0.003**
Lactates				
>5 (mmol/L) baseline	14 (13.9)	5 (17.2)	1.31 (0.43–3.99)	0.636
Increasing at progression	21 (20.8)	7 (24.1)	3.53 (1.42–7.87)	**0.049**
Echocardiography at diagnosis				
LVEF (%)	44.1 ± 10.7	39.2 ± 12.1	0.96 (0.92–1.0)	0.066
TAPSE (mm)	14.5 ± 4.6	11.6 ± 4.2	0.84 (0.72–0.98)	**0.026**
S’TDI (cm/s)	8.7 ± 3.6	6.0 ± 2.8	0.75 (0.52–1.09)	0.104
Biventricular Dysfunction	33 (32.7)	11 (37.9)	1.57 (0.60–4.11)	0.362

CPR: cardiopulmonary resuscitation; CA: cardiac arrest; PCI: percutaneous coronary intervention; SBP: systolic blood pressure; LVEF: left ventricular ejection fraction; TAPSE: tricuspid annular plane systolic excursion; and S’TDI: S’ tissue Doppler imaging. Statistically significant variables are highlighted with bold.

**Table 7:** In-hospital clinical characteristics and outcomes of worsening cardiogenic shock.

	Worsening *n* = 20	Non-Worsening *n* = 110	OR (CI)	*p*-Value
In-Hospital Clinical Characteristics				
Cardiac arrest	6 (30.0)	35 (31.8)	0.91 (0.33–2.59)	0.872
PCI failure	10 (50%)	17 (15.4)	5.41 (1.96–14.97)	**<0.001**
MCS				
VA-ECMO	4 (20.0)	5 (4.5)	5.25 (1.27–21.63)	**0.022**
IABP	5 (25.0)	36 (32.7)	0.68 (0.23–2.03)	0.494
Impella LV	5 (25.0)	4 (3.6)	8.83 (2.13–36.6)	**0.003**
Impella RP	1 (7.3)	8 (5.0)	0.67 (0.079–5.67)	0.713
Peak lactates (mmol/L)	9.05 [4.5–13.5]	3.60 [2.4–5.9]	1.15 (1.05–1-26)	**0.003**
Peak creatinine (mg/dL)	2.39 [1.3–3.7]	1.13 [0.9–1.8]	1.32 (1.02–1.70)	**0.031**
Peak AST (mU/mL)	411 [213–1713]	236 [162–362]	1.5 (1.1–3.2)	**0.019**
Peak ALT (mU/mL)	118 [54–698]	63 [40–109]	1.02 (1.01–1.09)	**0.043**
Outcomes				
Death	13 (65.0)	15 (13.6)	11.63 (4.0–33.87)	**<0.001**
Major bleeding	4 (20.0)	7 (6.4)	3.70 (2.2–14.0)	**0.044**
Sepsis	4 (20.0)	17 (15.5)	1.37 (0.41–4.59)	0.611
Device weaning failure	7 (35.0)	3 (2.7)	3.53 (1.16–11.2)	**0.032**
ICU stay (days)	5 [3–8]	4 [3–8]	0.97 (0.92–1.04)	0.484
Hospital stays (days)	7.5 [3.25–13.5]	10 [7–17]	0.97 (0.93–1.01)	0.263

PCI: percutaneous coronary intervention; VA-ECMO: venoarterial extracorporeal membrane oxygenation; IABP: intra-aortic balloon pump; AST: aspartate aminotransferase; ALT: alanine transaminase; and ICU: intensive care unit. Statistically significant variables are highlighted with bold.

## Data Availability

Information regarding the data is available upon reasonable request.

## Supplementary Materials

The following supporting information can be downloaded at https://www.mdpi.com/article/10.3390/jcm14072231/s1, Table S1. SCAI Stage definitions proposed by the CSWG and used in this study; Table S2. Comparison of baseline characteristic of survivors and non-survivors; Table S3. Comparison of baseline characteristic of worsening and non-worsening.

## Abbreviations

ACS	acute coronary syndrome (s)
AMICS	acute myocardial infarction with cardiogenic shock
CA	cardiac arrest
CAD	coronary artery disease
CMR	cardiac magnetic resonance
CPR	cardiopulmonary resuscitation
CS	cardiogenic shock
ICU	intensive care unit
HF	heart failure
LV	left ventricle/ventricular
LVEF	left ventricular ejection fraction
OHCA	out-of-hospital cardiac arrest
(p) PCI	(primary) percutaneous coronary intervention
RCT	randomized controlled trial
RV	right ventricle/ventricular
SBP	systolic blood pressure
SCAI	Society of Cardiovascular Angiography and Interventions
STEMI	ST-elevation myocardial infarction
TAPSE	tricuspid annular plane systolic excursion
WPs	worsening patients

## References

[1] Aissaoui N., Puymirat E., Delmas C., Ortuno S., Durand E., Bataille V., Drouet E., Bonello L., Bonnefoy-Cudraz E., Lesmeles G. (2020). Trends in cardiogenic shock complicating acute myocardial infarction. Eur. J. Heart Fail..

[2] Babaev A., Frederick P.D., Pasta D.J., Every N., Sichrovsky T., Hochman J.S., NRMI Investigators (2005). Trends in Management and Outcomes of Patients with Acute Myocardial Infarction Complicated by Cardiogenic Shock. JAMA.

[3] Kapur N.K., Kanwar M., Sinha S.S., Thayer K.L., Garan A.R., Hernandez-Montfort J., Zhang Y., Li B., Baca P., Dieng F. (2022). Criteria for Defining Stages of Cardiogenic Shock Severity. J. Am. Coll. Cardiol..

[4] Jeger R.V., Radovanovic D., Hunziker P.R., Pfisterer M.E., Stauffer J.-C., Erne P., Urban P., AMIS Plus Registry Investigators (2008). Ten-Year Trends in the Incidence and Treatment of Cardiogenic Shock. Ann. Intern. Med..

[5] van Diepen S., Katz J.N., Albert N.M., Henry T.D., Jacobs A.K., Kapur N.K., Kilic A., Menon V., Ohman E.M., Sweitzer N.K. (2017). Contemporary Management of Cardiogenic Shock: A Scientific Statement From the American Heart Association. Circulation.

[6] Gupta T., Weinreich M., Kolte D., Khera S., Villablanca P.A., Bortnick A.E., Wiley J.M., Menegus M.A., Kirtane A.J., Bhatt D.L. (2020). Comparison of Incidence and Outcomes of Cardiogenic Shock Complicating Posterior (Inferior) Versus Anterior ST-Elevation Myocardial Infarction. Am. J. Cardiol..

[7] Kapur N.K., Paruchuri V., Jagannathan A., Steinberg D., Chakrabarti A.K., Pinto D., Aghili N., Najjar S., Finley J., Orr N.M. (2013). Mechanical Circulatory Support for Right Ventricular Failure. JACC Heart Fail..

[8] Hochman J.S., Sleeper L.A., Webb J.G., Sanborn T.A., White H.D., Talley J.D., Christopher E.B., Jacobs A.K., Slater J.N., Col J. (1999). Early Revascularization in Acute Myocardial Infarction Complicated by Cardiogenic Shock. N. Engl. J. Med..

[9] Farooq V., van Klaveren D., Steyerberg E.W., Meliga E., Vergouwe Y., Chieffo A., Kappetein A.P., Colombo A., Holmes D.R., Mack M. (2013). Anatomical and clinical characteristics to guide decision making between coronary artery bypass surgery and percutaneous coronary intervention for individual patients: Development and validation of SYNTAX score II. Lancet.

[10] Naidu S.S., Baran D.A., Jentzer J.C., Hollenberg S.M., van Diepen S., Basir M.B., Grines C.L., Diercks D.B., Hall S., Kapur N.K. (2022). SCAI SHOCK Stage Classification Expert Consensus Update: A Review and Incorporation of Validation Studies. J. Am. Coll. Cardiol..

[11] Mehran R., Rao S.V., Bhatt D.L., Gibson C.M., Caixeta A., Eikelboom J., Kaul S., Wiviott S.D., Menon V., Nikolsky E. (2011). Standardized bleeding definitions for cardiovascular clinical trials: A consensus report from the Bleeding Academic Research Consortium. Circulation.

[12] Thiele H., Zeymer U., Akin I., Behnes M., Rassaf T., Mahabadi A.A., Lehmann R., Eitel I., Graf T., Seidler T. (2023). Extracorporeal Life Support in Infarct-Related Cardiogenic Shock. N. Engl. J. Med..

[13] Møller J.E., Engstrøm T., Jensen L.O., Eiskjær H., Mangner N., Polzin A., Schulze P.C., Skurk C., Nordbeck P., Clemmensen P. (2024). Microaxial Flow Pump or Standard Care in Infarct-Related Cardiogenic Shock. N. Engl. J. Med..

[14] Rudski L.G., Lai W.W., Afilalo J., Hua L., Handschumacher M.D., Chandrasekaran K., Solomon S.D., Louie E.K., Schiller N.B. (2010). Guidelines for the Echocardiographic Assessment of the Right Heart in Adults: A Report from the American Society of Echocardiography: Endorsed by the European Association of Echocardiography, a registered branch of the European Society of Cardiology, and the Canadian Society of Echocardiography. J. Am. Soc. Echocardiogr..

[15] Vakil D., Soto C., D’costa Z., Volk L., Kandasamy S., Iyer D., Ikegami H., Russo M.J., Lee L.Y., Lemaire A. (2021). Short-term and intermediate outcomes of cardiogenic shock and cardiac arrest patients supported by venoarterial extracorporeal membrane oxygenation. J. Cardiothorac. Surg..

[16] Konstam M.A., Kiernan M.S., Bernstein D., Bozkurt B., Jacob M., Kapur N.K., Kociol R.D., Lewis E.F., Mehra M.R., Pagani F.D. (2018). Evaluation and Management of Right-Sided Heart Failure: A Scientific Statement From the American Heart Association. Circulation.

[17] Bergamaschi L., Arangalage D., Maurizi N., Pizzi C., Valgimigli M., Iglesias J.F., Landi A., Leo L.A., Eeckhout E., Schwitter J. (2024). Hepatic T1 mapping as a novel cardio-hepatic axis imaging biomarker early after ST-elevation myocardial infarction. Eur. Heart J.-Cardiovasc. Imaging.

[18] Voelkel N.F., Quaife R.A., Leinwand L.A., Barst R.J., McGoon M.D., Meldrum D.R., Dupuis J., Long C.S., Rubin L.J., Smart F.W. (2006). Right Ventricular Function and Failure. Circulation.

[19] Gramegna M., Beneduce A., Bertoldi L.F., Pagnesi M., Marini C., Pazzanese V., Camici P.G., Chieffo A., Pappalardo F. (2020). Impella RP support in refractory right ventricular failure complicating acute myocardial infarction with unsuccessful right coronary artery revascularization. Int. J. Cardiol..

[20] Harjola V., Mebazaa A., Čelutkienė J., Bettex D., Bueno H., Chioncel O., Crespo-Leiro M.G., Falk V., Filippatos G., Gibbs S. (2016). Contemporary management of acute right ventricular failure: A statement from the Heart Failure Association and the Working Group on Pulmonary Circulation and Right Ventricular Function of the European Society of Cardiology. Eur. J. Heart Fail..

[21] Belletti A., Lerose C.C., Zangrillo A., Landoni G. (2021). Vasoactive-Inotropic Score: Evolution, Clinical Utility, and Pitfalls. J. Cardiothorac. Vasc. Anesth..

[22] Kanwar M.K., Everett K.D., Gulati G., Brener M.I., Kapur N.K. (2022). Epidemiology and management of right ventricular-predominant heart failure and shock in the cardiac intensive care unit. Eur. Heart J. Acute Cardiovasc. Care.

[23] Ostadal P., Rokyta R., Karasek J., Kruger A., Vondrakova D., Janotka M., Naar J., Smalcova J., Hubatova M., Hromadka M. (2023). Extracorporeal Membrane Oxygenation in the Therapy of Cardiogenic Shock: Results of the ECMO-CS Randomized Clinical Trial. Circulation.

[24] Baran D.A., Grines C.L., Bailey S., Burkhoff D., Hall S.A., Henry T.D., Hollenberg S.M., Kapur N.K., O’Neill W., Ornato J.P. (2019). SCAI clinical expert consensus statement on the classification of cardiogenic shock: This document was endorsed by the American College of Cardiology (ACC), the American Heart Association (AHA), the Society of Critical Care Medicine (SCCM), and the Society of Thoracic Surgeons (STS) in April 2019. Catheter. Cardiovasc. Interv..

[25] Chieffo A., Dudek D., Hassager C., Combes A., Gramegna M., Halvorsen S., Huber K., Kunadian V., Maly J., Møller J.E. (2021). Joint EAPCI/ACVC expert consensus document on percutaneous ventricular assist devices. Eur. Heart J. Acute Cardiovasc. Care.

[26] Spadafora L., Betti M., D’ascenzo F., De Ferrari G., De Filippo O., Gaudio C., Collet C., Sabouret P., Agostoni P., Zivelonghi C. (2025). Impact of In-Hospital Bleeding on Post-Discharge Therapies and Prognosis in Acute Coronary Syndromes. J. Cardiovasc. Pharmacol..

