# Supplementary material for: Patterns of Disease Progression and Outcomes of Inferior ST-Elevation Myocardial Infarction Complicated by Cardiogenic Shock: The Multicenter INSTINCT Registry
Source: J Clin Med. 2025 Mar 25;14(7):2231. doi: 10.3390/jcm14072231 (PMC11989967; doi:10.3390/jcm14072231)
Supplement: Supplementary file 1 [file jcm-14-02231-s001.zip › jcm-3473788-supplementary.pdf]

Supplementary Material

Supplementary Table S1. SCAI Stage definitions proposed by the CSWG and used in this study

SCAI Stage	Definition
SCAI B	Hypotension: SBP 60-90 mmHg or MAP 50-65mmHg
Beginning	OR
	Hypoperfusion: Lactates 2-5mmol/l or ALT 200-500mU/ml
	NO device or drug therapy
SCAI C	Hypotension: SBP 60-90 mmHg or MAP 50-65mmHg
Classic	AND
	Hypoperfusion: Lactates 2-5mmol/l or ALT 200-500mU/ml
	OR
	Need for 1 drug (Inotropic Score <10)/ 1 MCS device
SCAI D	Hypotension: SBP 60-90 mmHg or MAP 50-65mmHg
Deteriorating	AND
	Hypoperfusion: Lactates 5-10 mmHg or ALT>500mU/ml
	OR
	Need for 2-5 drugs (Inotropic Score 10-20) or MCS devices
SCAI E	Hypotension: SBP <60mmHg or MAP <50mmHg
Extreme	OR
	Hypoperfusion: Lactates >10mmol/l or pH ≤7.2
	OR
	Need for >3 drugs (Inotropic Score >20) or >3 devices

SCAI: Society for Cardiovascular Angiography and Interventions SBP: systolic blood pressure, MAP: mean arterial pressure, ALT: alanine transaminase, MCS: mechanical circulatory support

Supplementary Table S2. Comparison of baseline characteristic of survivors and non-survivors

	Survivors n=101	***Non survivors n=29***	***p-Value***
**Baseline Characteristics**			
Female gender	30 (29.7)	11 (37.9)	0.335
Age	68.1 ± 12.3	74.7 ± 8.5	0.020
BMI	26.8 ± 4.2	25.1 ± 3.7	0.069
Smoker	59 (58.4)	9 (31.0)	0.014
Diabetes	29 (28.7)	10 (34.4)	0.357
Hypertension	65 (64.3)	20 (68.9)	0.298
CAD	11 (11.0)	9 (31.0)	0.004
Heart failure	2 (1.9)	2 (6.8)	0.150
Previous AMI	8 (7.9)	5 (17.2)	0.097
Previous CABG	4 (4.0)	3 (10.3)	0.147
Previous PCI	9 (8.9)	7 (24.1)	0.018
Atrial fibrillation	7 (6.9)	4 (13.8)	0.194
COPD	13 (12.9)	4 (13.8)	0.792
CVA	8 (7.9)	3 (10.3)	0.599
CKD	27 (34.7)	11 (37.9)	0.209

BMI: body mass index, CAD: coronary artery disease, AMI: acute myocardial infarction, CABG: coronary artery bypass graft, PCI: percutaneous coronary intervention, COPD: chronic obstructive pulmonary disease, CVA: cerebrovascular accident, CKD: chronic kidney disease

Supplementary Table S3. Comparison of baseline characteristic of worsening and non-worsening patients

	**Worsening n=20**	**Non-worsening n=110**	***p-Value***
**Baseline Characteristics**			
Female gender	6 (30.0)	35 (31.8)	0.872
Age	73.0 ± 14.2	69.3 ± 12.0	0.221
BMI	27.6 ± 5.0	25.2 ± 3.6	0.035
Smoker	8 (40.0)	60 (54.5)	0.471
Diabetes	8 (40.0)	31 (28.1)	0.471
Hypertension	14 (70.0)	71 (64.5)	0.438
CAD	3 (15.0)	17 (15.5)	
Heart failure	1 (5.0)	3 (2.7)	0.556
Previous AMI	2 (10.0)	11 (10.0)	0.717
Previous CABG	0	7 (6.3)	0.258
Previous PCI	3 (15.0)	13 (11.8)	0.628
Atrial fibrillation	2 (10.0)	9 (8.1)	0.735
COPD	2 (10.0)	15 (13.6)	0.711
CVA	1 (5.0)	10 (9.1)	0.581
CKD	7 (35.0)	31 (28.2)	0.554

BMI: body mass index, CAD: coronary artery disease, AMI: acute myocardial infarction, CABG: coronary artery bypass graft, PCI: percutaneous coronary intervention, COPD: chronic obstructive pulmonary disease, CVA: cerebrovascular accident, CKD: chronic kidney disease
